# Dr. S. W. Darrow

**Published:** 1915-06

**Authors:** 


**Dr. S. W. Darrow,** Cleveland Homoeopathic 1877, died at his home in Brockport, N. Y., May 4.

